# Cutaneous melanoma follow-up: appropriateness of requests for ultrasound tests – the S.Gallicano National Referral Centre Experience

**DOI:** 10.1186/1756-9966-32-73

**Published:** 2013-10-09

**Authors:** Francesco M Solivetti, Fulvia Elia, Antonino Guerrisi, Flora Desiderio, Maria Giulia Santaguida, Isabella Sperduti, Claudia Cavallotti, Aldo Di Carlo

**Affiliations:** 1 Radiology Department, San Gallicano Dermatology Institute, Via Chianesi, 53-00144 Rome, Italy; 2 Department of Medical-Surgical Sciences and Biotechnologies, La Sapienza University of Rome, Latina, Italy; 3 Biostatistical Unit, Regina Elena National Cancer Institute, Rome, Italy; 4 Clinical Department, San Gallicano Dermatology Institute, Rome, Italy; 5 San Gallicano Dermatology Institute, Rome, Italy

**Keywords:** Cutaneous melanoma, Defensive medicine, Ultrasound test appropriateness

## Abstract

**Background:**

Cutaneous melanoma is a malignant neoplasm with a constantly increasing incidence, the prognosis of which is largely dependent on early diagnosis. The appropriateness of requests for ultrasound (US) tests during melanoma follow-up of patients referred to our institute was evaluated.

**Patients and methods:**

The requests for US tests of all patients referred to our institute over a four-month period were assessed. In order to correctly evaluate the appropriateness of requests, patients were split into two groups on the basis of melanoma thickness: > 1 mm (Group A) and < 1 mm (Group B).

**Results:**

546 patients were enrolled in our study out of a total of 1240 US tests performed. Out of 290 Group A patients, 104 patients (35%) did not meet the established congruity criteria. Group B was composed of 256 individuals, 92 patients (35.9%) of which were found to have at least one inappropriate request.

**Conclusion:**

In our study, more than 30% of the requests for US tests were found to be inappropriate, to the detriment of those with a real need for diagnostic testing. This lengthens waiting lists and it may also increase public healthcare costs. Therefore, it is mandatory to adopt new, widely accepted and easily applicable guidelines.

## Introduction

Cutaneous melanoma is a malignant neoplasm with a constantly increasing incidence [1], the prognosis of which is still largely dependent on early diagnosis, which becomes unfavorable at an advanced stage, regardless of treatment.

Therefore, melanoma follow-up requires periodical clinical and instrumental tests which ought to be performed with standardized protocols and at preset time intervals.

To this intent, many different solutions have been proposed although widely accepted international guidelines are still lacking. There are significant differences, as confirmed by a variety of national guidelines [2-6] whose practical application in the clinical field is sometimes limited because of poor compliance on the part of some doctors and patients. For this reason, widely accepted guidelines from the major international medical Societies to regulate work-up of diagnostic-instrumental testing are needed. This would lead to a reduction of the ever-increasing costs for the healthcare system.

As a consequence, requests for inappropriate diagnostic US tests during follow-up leads to a lengthening of waiting lists, as well as a reduction of availability of US tests for other important diseases, and first of all urgent tests.

Moreover, not only can the screening of patients with excised low-risk lesion be considered unnecessary, but also detrimental, because people suffer from more anxiety about their health and can enter an endless loop of overdiagnosis, and possibly undergo overtreatment, a process which does not promote health, but rather disease.

The aim of our study was to verify the appropriateness of requests for the melanoma follow-up US tests performed at our institute, a national public referral centre for dermatology and oncology.

## Patients and methods

The requests for US tests of all patients referred to our institute for follow-up of malignant cutaneous melanoma, over a four-month period from July to October 2012, were assessed. Only those patients with complete clinical records were enrolled in the study.

In order to obtain these data, a form was prepared in advance for each single patient (Additional file 1).

Patients were split into two different groups on the basis of melanoma thickness, that always proves critical, either > 1 mm (Group A) or < 1 mm (Group B).

However, in the second group, we only considered appropriate US requests for patients who meet one or more of the following criteria [7] or risk factors:

– Presence of ulceration

– Number of mitoses > than 1 per mm^2^

– Regression

– Multiple or familiar melanoma

– Positive sentinel lymph node and/or in transit or distant metastases

– Suspicious clinical data or instrumental reports.

The following were considered inadequate:

– Tests performed less than one month after an analogous or similar previous negative test, unless specific and motivated reasons were expressed in the request;

– Tests performed more than five years after initial diagnosis, without any evidence of recurrence of disease or new melanoma during this period [8];

– Tests relative to melanoma in situ or < 1 mm not meeting Group B inclusion criteria, as previously listed;

– Tests indicating lymph nodes stations at a non-plausible anatomical site of drainage, in the absence of any significant objective evidence.

Furthermore, before performing US tests, patients were asked to self-assess their approach to testing, with special attention to their mood (i.e. anxiety, mistrust), and also to its usefulness according to a VAS (Visive Analogic Scale) score ranging from 0 (excessive or inadequate) to 100 (very useful). These data were included in the form.

Finally, given the limitations associated with the frequent need for long-term planning of investigations, in relation to planned follow-up visits, we calculated the time interval between the date of request and the date on which it was actually performed.

About 10% of US requests examined were excluded from the study for incomplete clinical and instrumental data obtained.

### Statistical methodology

All results were reported with frequencies and medians; the associations were estimated using the Chi-squared test or Fisher’s Exact, when appropriate. The comparison between the two groups of interest was evaluated using the Mann–Whitney test. All the analyses were performed utilizing SPSS statistical software. (SPSS, Chicago, Il, U.S.A.; Version 20.0).

## Results

The final study population was composed of 546 patients, respectively 277 females (50.7%) and 269 males (49.3%). The length of follow-up of these patients was 37 months (median time), with a mean of 2.3 tests performed per individual patient. A total number of 1240 US tests were performed over four months. The cost of these exams, borne by the national health care system, amounted to 41,882 Euros. Out of 1240, 378 requests (30.5%) were inappropriate. Results related to tumor localization and final study population characteristics are extensively reported in Tables 1, 2.

**Table 1:** Results related to the melanoma, the requests and the US examinations in Patient Group A (melanoma thickness > 1 mm) and Group B (< 1 mm)

**Results**	**Group A *n *=290**	**Group B *n *=256**
	***N *(%)**	***N *(%)**
*Site of melanomas*	18 (6.2)	8 (3.1)
*Head-neck*	138 (47.6)	116 (45.3)
*Upper torso*	32 (11.0)	30 (11.7)
*Lower torso*	30 (10.3)	38 (14.8)
*Upper Limbs*	72 (24.8)	64 (25.0)
*Lower Limbs*		
*Sentinel Lymph node*	228 (82.0)	2 (0.8)
*Ulceration*	20 (6.97)	0 (0)
*Regression*	2 (0.7)	2 (10.8)
*Multiple melanoma*	40 (13.8)	0 (0)
*Familiarity*	4 (1.4)	0 (0)
*Mitosis*	10 (3.4)	0 (0)
*Urgent requests*	16 (65.5)	4 (1.6)
*Total US tests*	644	596
*Total unjustified US tests*	206 (32.0)*	172 (28.9)*
*Total cost (Euros)*	21902.8	19979.6
*Unjustified cost (Euros)*	6709.4 (30.6)**	5704 (28.5)**

Note. * Out of total tests ** Out of total cost.

**Table 2:** Characteristics of the final study population (n = 546) split into two groups [Group A (melanoma thickness > 1 mm) and Group B (< 1 mm)]

**Characteristics**	**Group A n = 290**	**Group B n = 256**	**P value**
*Sex*	n(%)	n(%)	**0.88**
*M*	148 (51.0)	129 (50.4)	
*F*	142 (49.0)	127 (49.6)	
	**Median (range)**	**Median (range)**	**P value**
*Age (years)*	58 (18–86)	52 (26–81)	0.004
*Waiting Time (months)*	28.7 (0–86)	7 (0–63)	0.05
*Time between US tests (months)*	12.4 (0–37)	9 (0–74)	< 0.0001
*US tests/patients (N)*	2 (0–5)	2 (0–6)	0.19

**Figure 1:**
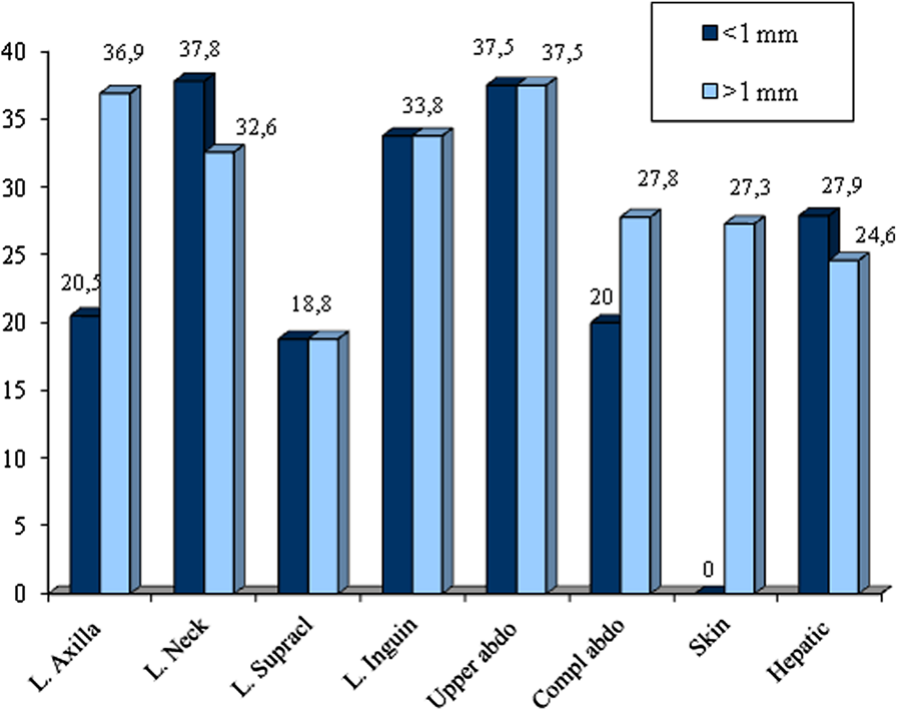
Inappropriated test according to tumor localization for patients of Group A and Group B.

Out of 546 patients, Group A comprised 290 individuals (53.1%) (melanoma thickness > 1 mm), and Group B comprised 256 individuals (46.9%) (melanoma thickness < 1 mm) (Figure 1).

In Group A, the median age was 58 years, while in Group B it was 52 years. Waiting time for Group A patients was 7 days on average, with a range of 0–63 days, whereas for Group B, average waiting time was 28.7 days, with a range of 0–86 days.

In the case of repeated tests, the interval between each test for Group A patients was 12.4 months on average , with a range of 0–37 months, whereas 9.3 months, with a range of 0–74 months was reported for Group B.

As for costs and test appropriateness: a total of 644 tests were performed in Group A (290 patients). In this group, 104 patients were found to have an inappropriate motivation (35.9%), for a total of 206 unjustified examinations (32%). Consequently, for this group there was a cost of 6,709 Euros for unjustified tests out of a total of 21,902 Euros.

596 tests were performed in Group B, formed of 256 individuals. In this group, 92 patients with at least one unjustified request (35.9%), and a total of 172 unjustified tests (29%) were reported. Consequently, 5,704 Euros was spent for unjustified tests out of a total cost of 19,976 Euros.

**Figure 2:**
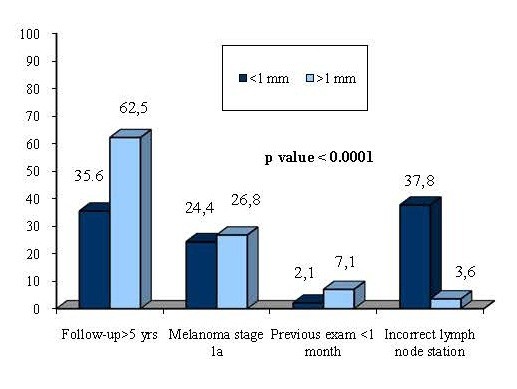
Reasons for Inappropriateness for patient both Group A and Group B.

It is interesting to note that the percentage of unjustified tests is similar in the two groups (32% for Group A vs. 29% for Group B, p = 0.53), although for different reasons. In fact, the most common among the unjustified requests in Group A was a test prescribed after more than 5 years (62.5%), whereas in Group B there were two main causes, the excessively long follow-up (35.6%) and incorrect indication of the lymph node station (37.8) (Figure 2).

Moreover, on the basis of patients’ perception, test usefulness was deemed very high since 97% of them expressed a satisfaction rate equivalent to the maximum VAS score.

In a subgroup of melanoma in situ (*N* = 81 patients, 13.5%), identified as part of Group B, further thorough exams were requested for 11 patients because of the incidental discovery of seven large hepatic angiomas, two adrenal adenomas, a complex renal cyst and a pancreatic pseudocyst, all irrelevant in relation to evolution of the clinical outcome as well as expensive for the national healthcare system and stressful for the patients. We found less percentages of “incidentalomas” in the other Subgroup B (5%) and Group A (12%).

## Discussion

According to our data, about 30% of US tests are inappropriate, which represents a high percentage if we consider these requests came from expert oncologists and dermatologists at our institute.

Since the total cost for US tests performed in our institute amounted to 41,882 Euros over a four-month period, the total cost per year could be estimated at 125,646 euro; of these, unjustified US tests had a charge of 12,413 Euros (6,709 Euros for Group A + 5704 Euros for Group B) for a four-month period, estimated at 37,239 Euros over a year (the unjustified expense for the institute is about the 30% of the total cost).

In the absence of other major studies, we know that in the year 2000 – the last available global data – the annual rate of US tests performed by Italian National Health Service facilities was 17.4 per 100 inhabitants [9]; consequently in order to evaluate such an economic burden for the whole country, we can estimate 30 million US tests performed per year (adding to them diagnostic tests carried out during hospitalization and by private health facilities, paid entirely by patients). This number is bound to increase in the following years, considering the further spread of the method and the improving technology that make it possible to include US tests in oncologic follow-up routines. If these values are related to the percentage of erroneous requests found in our study (about 30%), it is possible to assume that about 10,000,000 unnecessary U.S. tests may be performed in Italy per year.

They represent an enormous cost for our society which is no longer acceptable. It is also correct to say that an unjustified test could lead to further diagnostic tests which are not beneficial in relation to the underlying disease, and increase costs even more.

On the other hand, the appropriate use of complementary diagnostic tests during follow-up for melanoma could reduce costs related to patient management for this disease [10].

The relevant percentage of mistakes in identifying the lymph node station, that in our case studies shows an error rate of 32% for lesions of thickness > 1 mm and 29% for those < 1 mm [11], should also be underlined. The percentage of error is greater for the numerous requests for examination of multiple stations. They are certainly greater in number than those correctly examined, due to the practice of “defensive medicine”, which is the main cause of too long, if not totally unnecessary follow-ups, such as for melanomas in situ - stage 1a.

The waiting list in our institute is much shorter than the national one, the data obtained from our series is marred by an intrinsic enrollment bias; in fact, the requests for US tests are often spontaneously postponed by the patient, or sometimes also by the doctor who defers them until the scheduled oncological follow-up. However, it must be stressed that the need to meet all these inappropriate demands unfortunately results in a lengthening of waiting lists for other patients with obvious repercussions on public health.

From a patient viewpoint, it is important to underline the great degree of satisfaction of patients undergoing US tests for follow-up and the placebo effect generated by a favorable prognosis. The majority of these patients, regardless of the initial diagnosis, considered the examination of the utmost importance and were determined to undergo it even if their personal approach to the test was characterized by different moods; only 3% of patients considered follow-up imaging no longer useful and reported their unwillingness to undergo clinical check-ups because of a deep state of anxiety. Although there are no studies in this direction, the damage caused to patients as a result of unnecessary testing should be emphasized; harm related to the loss of working days, transfer expenses, and especially stress related to the expectation and execution of the examination. The same stress may also be the indirect cause of the high value assigned by our oncological patients to the examination itself, thus triggering dangerous feedback.

In our opinion this problem could be significantly reduced by creating new referral pathology centers where physicians are able to carry out correct work-up of the patients, share clinical data and establish complete computerized centralization of requests with an updated list of all the diagnostic, as well as therapeutic procedures performed, along with their final outcome also in order to reduce unnecessary/harmful repetition of the same diagnostic tests. Furthermore, we hope for strict compliance with existing guidelines, or the creation of new, universally accepted guidelines that provide better clinical and legal justification of the timing and nature of diagnostic tests required for the follow-up of melanoma, and consequently reduce the problem of defensive medicine emerging in Italy’s medical-legal framework.

## Conclusion

To conclude, it is clear that about 30% of the US diagnostic examinations performed are unjustified according to the general guidelines currently in use. Therefore, they have not been requested according to strict clinical scientific parameters, but for other reasons, possibly medical-legal ones.

Thus, there is need for the adoption of new shared, widely accepted and easily applicable guidelines, also in light of other considerations related to health costs and medical-legal aspects.

Given that said issues represent a thorny issue for other referral centers, we consider it absolutely necessary to update existing guidelines to make for easier use by specialists as well as General Practitioners.

## Competing interests

The authors declare that they have no competing interests.

## Authors’ contributions

MGS and IS have developed the statistical work; FMS devised the work have coordinated and have performed diagnostic tests; FE has performed diagnostic testing and data acquisition; AG, FD and CC participated in the drafting of labor, acquisition data and bibliography; Prof ADC as scientific director has coordinated and approved the work. All authors read and approved the final manuscript.

## Supplementary Material

Additional file 1 Form. Click here for file
