# Supplementary figures and images for: Cutaneous melanoma follow-up: appropriateness of requests for ultrasound tests – the S.Gallicano National Referral Centre Experience
Source: J Exp Clin Cancer Res. 2013 Oct 9;32(1):73. doi: 10.1186/1756-9966-32-73 (PMC3851827; doi:10.1186/1756-9966-32-73)

Additional file 1 **Form**


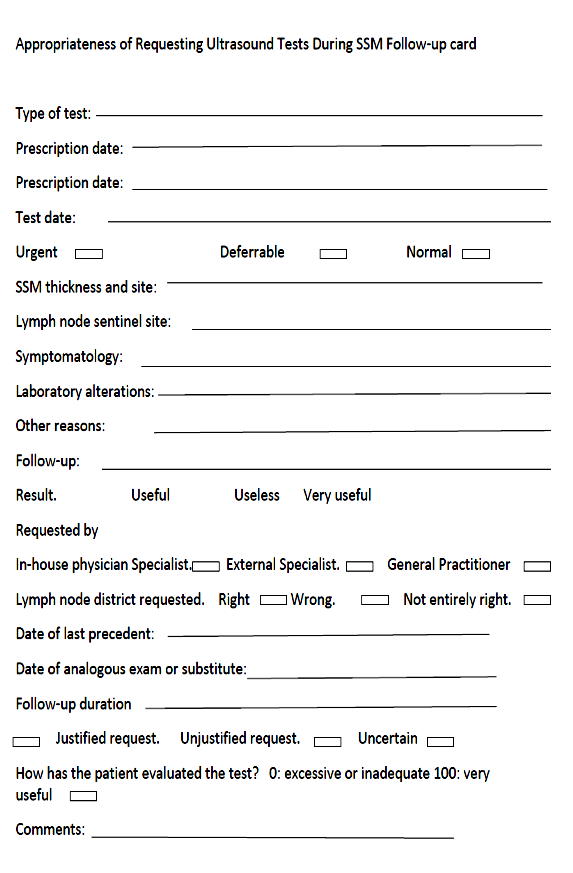

Supplement: Additional file 1 — Form. [file 1756-9966-32-73-S1.doc]
